# A systems biology approach to defining regulatory mechanisms for cartilage and tendon cell phenotypes

**DOI:** 10.1038/srep33956

**Published:** 2016-09-27

**Authors:** A. J. Mueller, S. R. Tew, O. Vasieva, P. D. Clegg, E. G. Canty-Laird

**Affiliations:** 1 Department of Musculoskeletal Biology, Institute of Ageing and Chronic Disease, Faculty of Health & Life Sciences, University of Liverpool, William Henry Duncan Building, 6 West Derby Street, Liverpool, L7 8TX, United Kingdom; 2 The MRC-Arthritis Research UK Centre for Integrated Research into Musculoskeletal Ageing (CIMA); 3 Institute of Integrative Biology, Biosciences Building, University of Liverpool, Crown St., Liverpool, L69 7ZB, United Kingdom

## Abstract

Phenotypic plasticity of adult somatic cells has provided emerging avenues for the development of regenerative therapeutics. In musculoskeletal biology the mechanistic regulatory networks of genes governing the phenotypic plasticity of cartilage and tendon cells has not been considered systematically. Additionally, a lack of strategies to effectively reproduce *in vitro* functional models of cartilage and tendon is retarding progress in this field. De- and redifferentiation represent phenotypic transitions that may contribute to loss of function in ageing musculoskeletal tissues. Applying a systems biology network analysis approach to global gene expression profiles derived from common *in vitro* culture systems (monolayer and three-dimensional cultures) this study demonstrates common regulatory mechanisms governing de- and redifferentiation transitions in cartilage and tendon cells. Furthermore, evidence of convergence of gene expression profiles during monolayer expansion of cartilage and tendon cells, and the expression of key developmental markers, challenges the physiological relevance of this culture system. The study also suggests that oxidative stress and PI3K signalling pathways are key modulators of *in vitro* phenotypes for cells of musculoskeletal origin.

The musculoskeletal system consists of a diverse group of tissues that facilitate movement and are developmentally, anatomically, and functionally interrelated. Of the musculoskeletal tissues, cartilage and tendon progenitor cells arise from common mesenchymal precursors with variable expression of *Sox9* (SRY (Sex Determining Region Y)-Box 9), and scleraxis (*Scx*), a basic helix-loop-helix (bHLH) transcription factor^1^,^2^. Furthermore, both tissues are comprised of highly specialized extracellular matrices with distinct architecture, are relatively acellular, avascular, and aneural with quiescent resident cell populations in physiological states. Both tissues show insidious degeneration with age and represent highly prevalent co-morbidities within ageing populations^3^.

In general, within the field of musculoskeletal biology, there has been little attention given to comparative studies of the constituent tissues of this system. The highly specialized three-dimensional structures inherent to the function of cartilage and tendon have yet to be adequately modelled *in vitro*^4^,^5^. Consequently, extrapolation of findings and translation to therapies is limited for these tissues. The dearth of disease modulating therapeutics may, in part, be associated with a deficiency of available *in vitro* models.

Resident tissue progenitor cells are reported to be isolated from both cartilage^6^ and tendon^7^. Additionally, methods to manipulate the phenotype of adult somatic cells^8^ have shown that the differentiated state is reversible and degrees of phenotypic plasticity exist. Terms including de-, re-, and trans-differentiation represent alterations in the synthetic profile of adult somatic cells that may be relevant to tissue regeneration^9^. Dedifferentiation, a loss of cellular functionality, is a term with no definitive mechanism, yet is used to describe distinct and diverse biological contexts^10^,^11^, including the response to monolayer expansion of chondrocytes^12^ and tenocytes^13^. Recent evidence also implicates the loss of differentiated (functional) status as a factor in degenerative disease with the inference that dedifferentiation of chondrocytes has some comparison with osteoarthritis pathways^14^. Consequently, exploration of mechanisms that may contribute to a deeper understanding of degenerative and regenerative phenotypes could help elucidate points for therapeutic intervention in conditions such as osteoarthritis and tendinopathy.

Biological phenomena, such as development, are complex dynamic processes. They cannot be reduced to the characteristics of their components and need to be described by interactions within multiple hierarchical levels comprising a system. Systems bioinformatics approaches, such as gene co-expression network reconstruction^15^ and pathway analysis^16^, facilitate a high-level understanding of a system under investigation.

A major obstacle to the development of *in vitro* cartilage models and therapeutic interventions is the capacity to control differentiation^5^. The plethora of biomolecules known to modulate the chondrocyte phenotype^14^ and the dynamic and transient nature of regulator expression, e.g. TGF-β1 oscillations^17^, suggest that manipulating differentiation through the application of a small number of growth factors with an invariant temporal profile is unlikely to be successful^5^. Systems biology approaches are well-suited to tackling the complexity of such relationships.

This study revisits the dedifferentiation/redifferentiation paradigm in chondrocytes using a sytems biology based approach and compares these processes with those occurring in tenocytes subjected to similar conditions. Using transcriptomic profiling and systems biology techniques the objective was to provide a clear hypothetical mechanistic description of the impact that *in vitro* environmental perturbations have on the system and evaluate the adequacy of three-dimensional *in vitro* culture models as proxies for cartilage and tendon. We hypothesised that the regulatory mechanisms that governed phenotypes in cartilage and tendon cells would be comparable in standard three-dimensional culture models. By establishing standardised characteristics for *in vitro* models, proposing regulatory mechanisms, and defining core differences between the cell types, step-wise improvements in the differentiated status of *in vitro* models may be achieved through further investigations.

## Results

### Dedifferentiated cells show convergence of gene expression profiles

**Figure 1:**
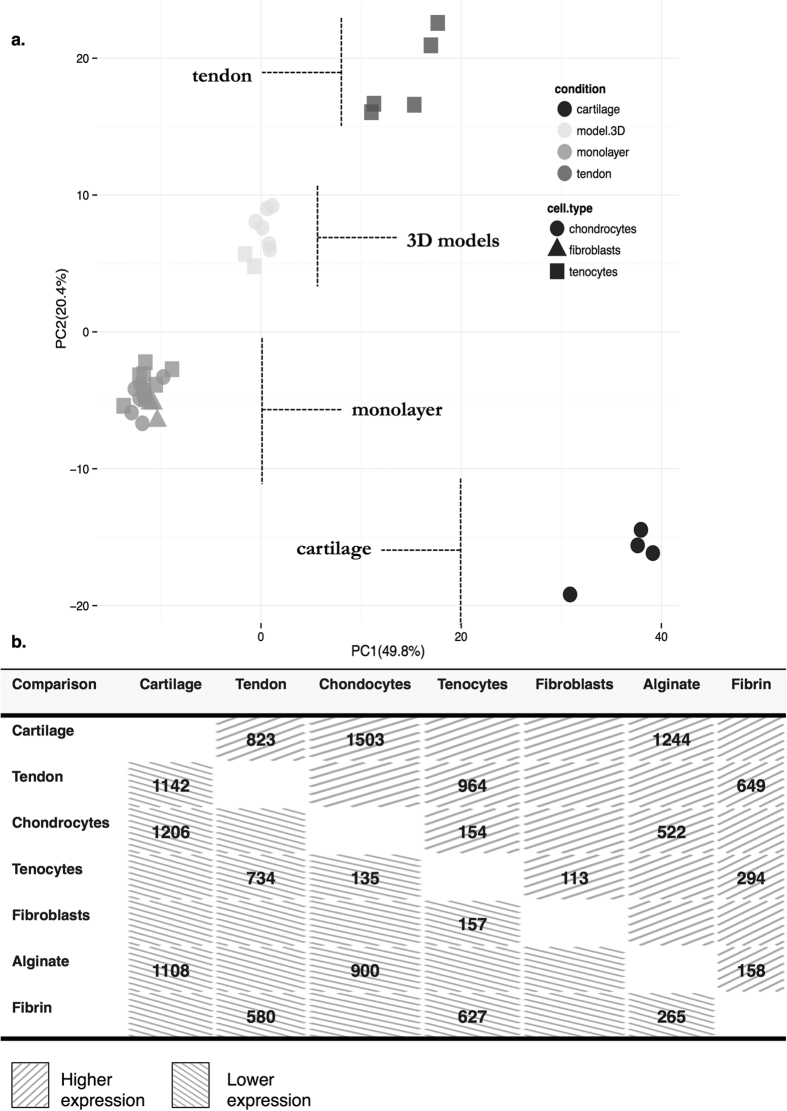
(**a**) Principal component analysis of gene expression data from 36 Illumina arrays profiling three cell types (cell type) and isolated from three environmental conditions (condition) – native (cartilage or tendon tissue), monolayer (passage 5, dedifferentiated), or 3D (alginate or fibrin cultures). The figure demononstrates the clustering of samples using the first two principal components (PC1, PC2), which together explain >70% of the variation in the data for the top 500 most covariant genes, Supplementary Data, SD1. Covariance is used to measure how random genes change with respect to one another. The figure demonstrates that the genes showing the greatest covariance explain the majority of the differences observed between the experimental groups. Cells in monolayer, derived from cartilage, tendon, or dermal fibroblasts, group when only the most covariant genes are considered. Chondrocytes and tenocytes in three-dimensional cultures also group together, but not with their tissues of origin. Overall, there were fewer genes differentially expressed between native tendon and 3D fibrin cultures than between native cartilage and 3D alginate cultures. This greater complexity in the synthetic profile of cartilage is indicated by the clustering of cartilage samples distant from all other samples. (**b**) Matrix of up- and down-regulated genes for selected pairwise comparisons involving different environmental conditions for chondrocytes and tenocytes. Values indicate the number of up- or down- regulated genes with a log_2_ fold change >+/−0.5, FDR <0.01 and a log-odds ratio of expression >0 (~50%). Duplicate Entrez gene identifiers are removed. The fewest differentially expressed genes were found between cultured cells, for example only 154 genes were more highly expressed in chondrocytes relative to monolayer tenocytes; 135 genes had lower expression in chondrocytes than tenocytes (289 differentially expressed in total). Number of genes showing higher expression (positive log_2_ fold-change, upward hatch) and lower expression (negative log_2_ fold-change, downward hatch) in each comparison are indicated.

Using the top 500 genes showing the greatest covariance in expression (Supplementary Data, SD1) 36 samples could be clustered into four distinct groups by principal component analysis (PCA), Fig. 1a. The first two principal components described 70.2% of the variation in the data. These groups consisted of: (i) native cartilage; (ii) native tendon; (iii) all monolayer cultured chondrocytes, tenocytes, and fibroblasts; (iv) or three-dimensional culture systems (alginate or fibrin cultures). Overall, few genes were differentially expressed between cell types in monolayer at passage five or between three-dimensional culture conditions, Fig. 1b. The variation in the gene expression was suggestive of a convergence of expression profiles for cells in monolayer and a failure of chondrocytes or tenocytes in three-dimensional culture to recapitulate differentiation status to parity with native tissue.

### Gene ontology descriptions

Dedifferentiated (monolayer) chondrocytes and tenocytes demonstrated frequent overlap in enriched biological process annotations. Complete annotations are available (Supplementary Data, SD2–SD7) with summary terms provided in Table 1. Shared functional terms for dedifferentiation were associated with: metabolism, development, extracellular matrix organisation, and redox balance. Response to oxidative stress, apoptosis, and metabolic process terms were significantly associated with genes more highly expressed in chondrocytes and tenocytes in three-dimensional cultures. Developmental terms relating to ‘vasculature development’, ‘cardiovascular development’, ‘post-embryonic development’ and ‘regulation of anatomical structure morphogenesis’ were defined for chondrocytes in alginate bead cultures and ‘collagen catabolic process’ for tenocytes in fibrin constructs.

### Native tissues exhibit complex and distinct transcriptomic profiles

#### Cartilage

Relative to monolayer-expanded chondrocytes, cartilage in adult rats was characterised by higher expression of collagen type II (*Col2a1*), aggrecan (*Acan*), thrombospondin 4 (*Thbs4*), chondroadherin (*Chad*), alkaline phosphatase (*Alpl*), clusterin (*Clu*), dentin matrix acidic phosphoprotein (*Dmp1*), and integrin-binding sialoprotein (*Ibsp*). Additionally, CCN-family genes, proteoglycans, cathepsins, chemokines, homeodomain genes, innate immune-associated elements and cytoskeletal-associated kinesins and tubulins were strongly represented in cartilage relative to monolayer chondrocytes, Table 2. Comprehensive tables of differentially expressed genes for all conditions are found in Supplementary Data (SD8–SD17).

#### Tendon

Troponins and myosins were the most highly expressed gene group in native tendon; specifically troponin I type 2 (*Tnni2*), actin α1 (*Acta1*) and creatine kinase (*Ckm*) had higher expression in native tendon relative to monolayer. Tenomodulin (*Tnmd*), mustang (*Mustn1*), elastin (*Eln*), keratocan (*Kera*), lubricin (*Prg4*), dermatopontin (*Dpt*), and apolipoprotein (*ApoE*) were more highly expressed in native tendon than in monolayer. Relative to native cartilage, tendon exhibited higher expression of *Serpinf1*, *Myod1*, *Igfbp6*, *Thbs4*, and clusterin (*Clu*). There were fewer differentially expressed genes between tendon and 3D cultures than cartilage and 3D cultures indicating a difference in the complexity of the synthetic profiles between the two tissues, Fig. 1b.

### Dedifferentiated cells express developmental markers

The fewest statistically significant genes were found between monolayer chondrocytes and tenocytes (Fig. 1b) with a number of genes highly expressed by both in monolayer. At passage five monolayer cells demonstrated higher expression of mesenchymal markers *Thy1* (also known as CD90) and prion protein encoding gene, *Prnp*, epithelial-mesenchymal transition regulator *Snai1*, and bHLH transcription factor *Twist1*. The TGF-β signalling elements *Tgfb2*, *Tgfb3*, *Smad6* and inhibitor *Smad7*, were also more highly expressed in monolayer culture, Table 2. Development-associated homeobox genes, encoding homeodomain proteins, expressed more highly in monolayer chondrocytes included *Pitx1*, a hind-limb coding gene, and *Prrx2*, a differentiation-associated homeobox gene. Wnt-signalling frizzled family receptors *Fzd1*, *Fzd2* and *Fzd8* were more highly expressed in monolayer chondrocytes than cartilage, except Wnt-signalling modulator *Frzb*, which demonstrated the reciprocal relationship. Dedifferentiated tenocytes expressed higher levels of genes associated with development, transcription factors, serine protease *Serpine1*, biglycan (*Bgn*), and the homolog of *slit* (*Drosophila*), *Slit3.*

### Three-dimensional cultures do not restitute dedifferentiated cells

There were 283 genes found to be more highly expressed in three-dimensional culture systems relative to native tissue, i.e. common to alginate and fibrin cultures, Fig. 1b. In three-dimensional cultures *Fos* and *Junb*, the components of the AP-1 heterodimeric transcription factor, the transmembrane glycoprotein osteoactivin gene *Gpnmb*, clusterin (*Clu*) and the bone morphogenetic protein receptor, type 1a (*Bmpr1a*) were all more highly expressed. Consistent with the gene ontology annotations there was upregulation of genes associated with oxidative stress (*Nfe2l2*), hypoxia (*Hif1a*) and anti-oxidant responses (*Sod2*, *Hmox1*) in both 3D culture systems. The transcription factor *Vegfb*, chemokine ligand 1 *Cxcl1*, and prostaglandin-endoperoxide synthase 2 (*Ptgs2*) were also more highly expressed in both culture systems.

Redifferentiating chondrocytes exhibited higher expression of immune-associated genes including cytokines, chemokines, and alarmin genes, as well as a number of chondrogenesis-associated genes, Table 2. Tenocytes in fibrin cultures, when compared to native tendon, expressed higher levels of metallothionein 1a (*Mt1a*), the BMP-antagonist and tendon development gene gremlin 1 (*Grem1*), and enolase 2 (*Eno2*), a neuron-associated enolase isoenzyme. The tenascin N/W isoform, *Tnn*, was highly expressed in fibrin cultures.

### Validation of differentially expressed genes by qPCR

Genes chosen for corroboration of expression by qPCR were either common to culture systems, known or putative markers of developmental or differentiated musculoskeletal tissue. Genes with established differentiation roles in cartilage (*Sox9*) and tendon (*Scx*, *Mkx*) were also assessed although not found to be differentially expressed in microarray analysis.

**Figure 2:**
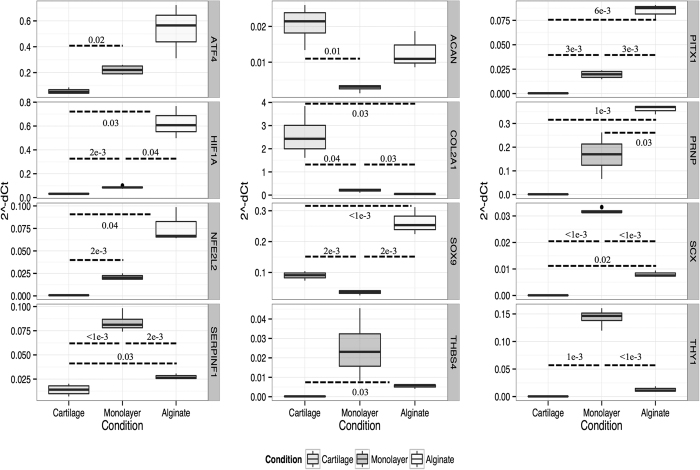
Box-and-whisker plots showing the distribution of normalised and transformed cycle threshold qPCR values (*y-axis*, 2^-dCt) for three experimental conditions (*x-axis*, cartilage | monolayer | alginate) for selected genes (right side vertical legend). Box and whisker plots show mean, first and third quartiles, and maximum and minimum values. Results shown are for technical triplicates on n = 4 (cartilage, monolayer) or n = 3 (alginate) biological replicates. Results of hypothesis testing (*p*-values) for differences between groups, after *post-hoc* corrections, are shown where thresholds are met.

**Figure 3:**
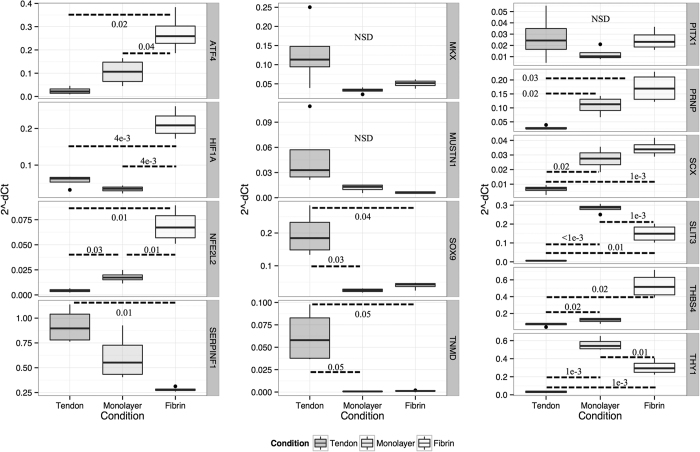
Box-and-whisker plots showing the distribution of normalised and transformed cycle threshold qPCR values (*y-axis*, 2^-dCt) for three experimental conditions (*x-axis*, tendon | monolayer | fibrin cultures) for selected genes (vertical legends). Box and whisker plots show mean, first and third quartiles, and maximum and minimum values. Results for technical triplicates on n = 4 biological replicates. Results of hypothesis testing (*p*-values) for differences between groups, after *post-hoc* corrections, are shown where thresholds are met; NSD – no significant difference.

Significant reductions in the expression of *Col2a1, Acan*, and *Sox9* for dedifferentiated chondrocytes, Fig. 2, and *Tnmd* and *Serpinf1* for dedifferentiated tenocytes, Fig. 3, were confirmed. The higher expression of *Thbs4* in cartilage relative to monolayer chondrocytes was not confirmed by qPCR. Both dedifferentiated chondrocytes and tenocytes were shown to have elevated expression of scleraxis (*Scx*), prion (*Prnp*) and *Thy1* and a reduction in *Sox9* expression. Alginate and fibrin 3D cultures expressed significantly higher levels of *Hif1a* and *Nfe2l2*, relative to native tendon or cartilage. Fibrin cultures significantly elevated expression of *Atf4*; a comparable trend was observed in alginate cultures. Alginate cultures exhibited higher expression of *Pitx1* and *Sox9*, whilst fibrin cultures up-regulated *Thbs4, Scx,* and *Slit3*. Expression of tissue-specific differentiation markers (*Tnmd, Col2a1*) was not restored in three-dimensional cultures.

### Pathway topology analysis defines consensus canonical pathways

**Figure 4:**
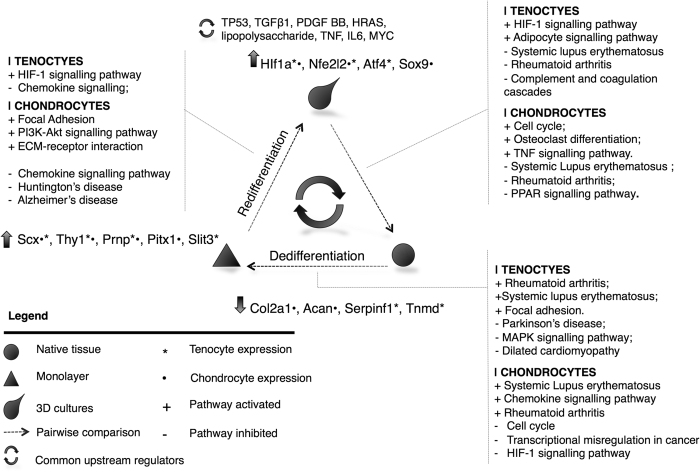
Schematic representation of gene expression profile comparisons made between each experimental condition and cell type. Each comparison (legend) is annotated with the most significant KEGG reference pathways and the activation status of that pathway as predicted using a Signalling Pathway Topology Analysis (SPIA), Supplementary Data, SD18,19. For each comparison and cell type the trend in expression of genes validated by qPCR is indicated, for example, in dedifferentiation a reduction in the expression of *Col2a1* and *Acan* is found in chondrocytes. Common upstream master regulators predicted to be associated with the observed gene expression profiles, from Ingenuity^®^ Pathway Analysis, are also provided.

Signalling Pathway Impact Analysis (SPIA), which uses a combination of over-expression analysis of differentially expressed gene lists, magnitude of fold-change, and the topology of KEGG canonical signalling pathways to define global pathway perturbation scores, predicted activation of ‘focal adhesion’, ‘rheumatoid arthritis’ and ‘systemic lupus erythematosus’ reference pathways in both native tissue to monolayer comparisons, Fig. 4. The HIF-1 and PI3K signalling pathways were predicted to be reciprocally inhibited or activated in de- and redifferentiation, respectively, for both cell types, see Supplementary Data (SD18–19). Inhibition of the ‘chemokine signalling pathway’ was common to both chondrocytes and tenocytes cultivated as a monolayer in comparison to those in three-dimensional culture systems. Alginate and fibrin cultures were shown to have significant activation of ‘osteoclast differentiation’ and ‘HIF1 signalling’ pathways, respectively. These perturbed pathways were consistent with the gene ontology functional annotations.

### Mechanistic networks for de- and redifferentiation transitions

**Figure 5:**
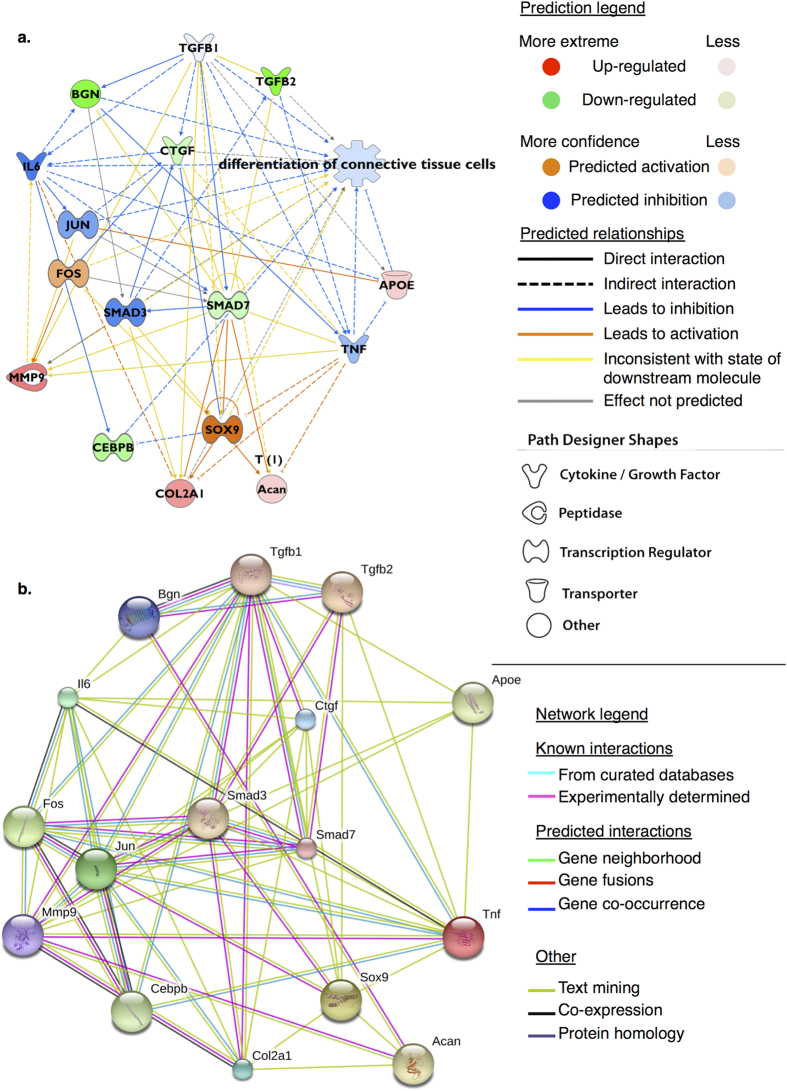
(**a**) Hypothetical mechanistic network derived from differentially expressed gene expression profile for cartilage versus dedifferentiated chondrocytes (dedifferentiation transition). Network consists of genes (nodes) connected by lines (edges) indicating a known relationship/interaction in the IPA^®^ Knowledge Base. Genes more highly expressed in cartilage are coloured red; genes showing low expression in cartilage (i.e. higher in monolayer) are coloured green. Figure prediction legend describes the nature of edges joining nodes. Upstream regulators, and intermediate nodes (*Jun, Smad3, Il6, Tnf*), were predicted to be activated (orange) or inhibited (blue) consistent with the gene expression profile supplied; the direction and nature of the relationship is also indicated. Based upon the observed gene expression profile *Tgf-β1* was predicted to be a key upstream regulator (z-score -2.65, overlap *p*-value 5.41e-26) of 740 differentially expressed genes; the actions of *Tgf-β1* were predicted to be inhibited in native cartilage where expression of *Smad7*, *Bgn,* and *Ctgf* were low relative to monolayer chondrocytes at passage five. Functional annotations for ‘differentiation of chondrocytes’ (3.66e-11), and ‘differentiation of connective tissue cells’ (5.6e-21, inhibited) were significantly enriched for this subnetwork and indicated a differentiation process; (**b**) Using the same elements a protein-protein association network consisting of nodes (proteins) and edges (evidence of associations) indicates a shared function between selected nodes, but not necessarily physical interactions. Sources for evidence of associations are defined in the network legend. Elements determined to influence differentiation status of chondrocytes and tenocytes in culture using IPA^®^ are shown to have significant enrichment for protein-protein interactions (*p* < 0.0001) indicating that as a group they are biologically connected.

To develop testable hypothetical mechanistic networks for de- and redifferentiation, upstream master regulators of the observed gene expression profiles for all pairwise comparisons, were inferred using Ingenuity^®^ Pathway Analysis (IPA). The top scoring upstream regulators were ordered by ‘overlap *p*-value’, a measure of the enrichment of regulated genes within a data set, and by calculated ‘*z*-score’ for the predicted activation state of a regulator inferred from a test of the match in up- and down-regulation patterns. In all comparisons the top predictions for upstream regulators of the observed synthetic profile were TGF-β, TNF, and MYC. For the dedifferentiation transition in both chondrocytes, Fig. 5a,b, and tenocytes TGF-β mediated effects were predicted to be inhibited in native tissue, i.e. activated in dedifferentiation. Additionally, IL6 was predicted for chondrocytes and HIF1α and PDGF BB for tenocytes.

**Figure 6:**
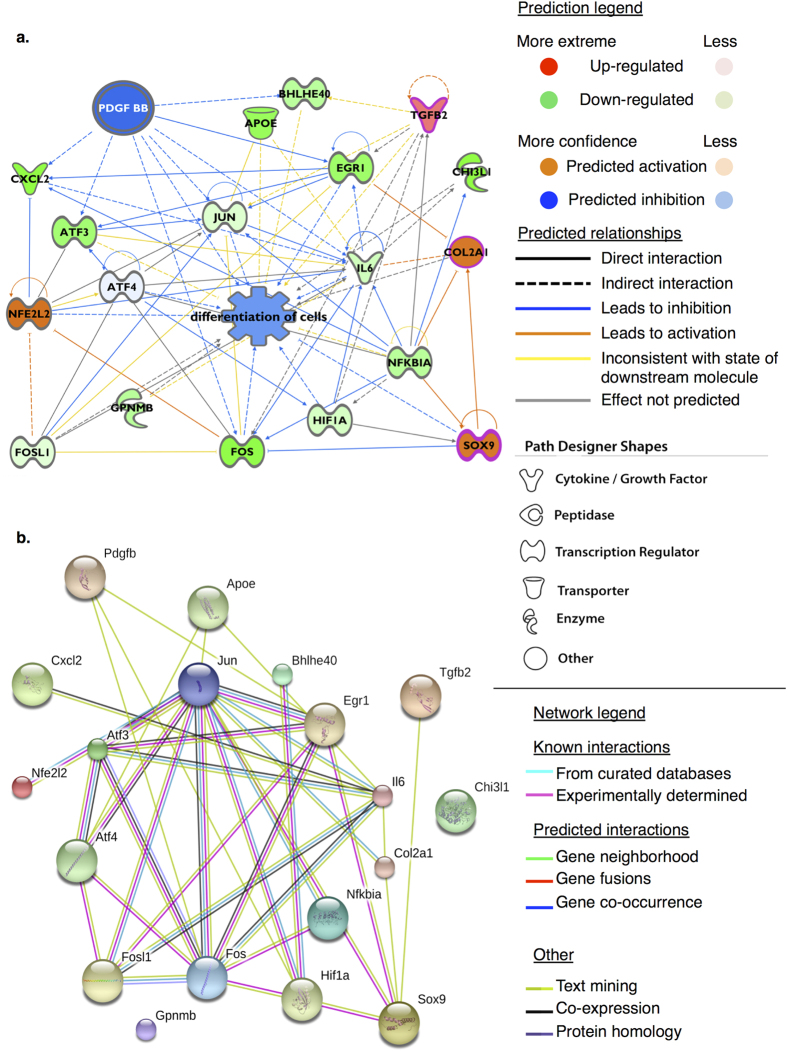
(**a**) Hypothetical mechanistic network derived from predicted upstream regulators for genes found to be differentially expression in chondrocytes in transition from monolayer to alginate bead cultures (redifferentiation). Genes showing lower expression in monolayer relative to alginate beads (*Hif1a, Nfe2l2, Atf3*) are coloured green (i.e. indicating higher expression in alginate cultures); genes in orange are predicted. Genes more highly expressed in monolayer are not shown (absence of red-coloured genes). In monolayer chondrocytes PDGF BB mediated effects are predicted to be inactive for the observed expression profile, i.e. activated in alginate cultures. Ingenuity^®^ canonical pathways significantly enriched for this expression profile included ‘NRF2-mediated oxidative stress’, ‘PI3K/AKT signalling’ and ‘the role of osteoblasts, osteoclasts and chondrocytes in rheumatoid arthritis’. The physiological function terms ‘differentiation of cells’ (*p* = 1.6e-14) and ‘apoptosis’ (*p* = 4.9e-12) were highly enriched in this subnetwork. Nodes with a pink border were enriched for ‘condensation of cartilage tissue’, *p* = 2.07e-7. The small molecule inhibitor of the PI3K/AKT pathway ‘LY294004’ was predicted to be activated based upon the supplied gene expression profile (z-score = 4.58, overlap *p*-value = 3.3e-15, 219 gene interactions). This mechanistic network indicates that PI3K signalling and PDGF BB are likely to be active in the redifferentiation phenotype in alginate cultures; the PI3K inhibitor LY294004 describes the inverse for monolayer cells. (**b**) Using the same elements a protein-protein association network consisting of nodes (proteins) and edges (evidence of associations) indicates a shared function between selected nodes, but not necessarily physical interactions. Sources for associations are defined in the network legend. Elements determined to influence differentiation status of chondrocytes and tenocytes in culture are shown to have significant enrichment for protein-protein interactions (*p* < 0.0001) indicating that as a group they are biologically connected. Some elements did not demonstrate evidence of association in this network (GPNMB, CHI3L).

Further evidence of consensus for upstream regulators was evident in analysis of the expression data for the transition between two- and three-dimensional culture systems. For both alginate, Fig. 6a,b, and fibrin cultures the gene expression profiles were consistent with PDGF BB and TNF as activated upstream regulators. Reciprocal activation was predicted for the small molecule inhibitor of PI3K signalling, LY294004, and PI3K activator PDGF BB in 3D cultures, consistent with SPIA prediction of activation of the PI3K pathway in redifferentiation.

## Discussion

The study sought to define testable mechanistic networks for de- and redifferentiation to establish the framework for iterative systems analysis, quantitative modelling, and to drive rational improvements in cell culture models for cartilage and tendon. This was based upon the hypothesis that comparable mechanisms regulated de- and re-differentiation in both cell types. The objectives of the study contributed to the wider goal of defining gene regulatory networks that may contribute to degenerative phenotypes in cartilage and tendon and, therefore, inform evidence-based development of organotypic culture systems in tissue engineering. Exploiting any inherent plasticity in musculoskeletal cells for regenerative purposes may be possible through a deeper understanding of these mechanisms. Although dedifferentiation of chondrocytes in monolayer culture has been an observed phenomenon for over 30 years^12^ this is the first comprehensive profiling and systematic comparison of musculoskeletal cells and mechanistic interpretation of this phenotype with a view to establishing systems models for de- and re-differentiation as potential mechanisms in regenerative therapy for cartilage and tendon.

A striking finding of this study was the phenotypic drift of musculoskeletal cells in monolayer culture and convergence of the gene expression profiles for chondrocytes, tenocytes, and dermal fibroblasts at passage five. Fewer than three-hundred genes were found to be differentially expressed between dedifferentiated chondrocytes and tenocytes. Of the most highly differentially expressed, only twenty-five had a log_2_ fold-change greater than two. Despite monolayer culture representing a fundamental research tool and pre-requisite for autologous cell therapy^18^ there is little scrutiny or comparison of gene expression profiles across musculoskeletal cell types. Given the reduction in the complexity of the monolayer environment from tissue this finding is perhaps not surprising. Although this phenomenon has not been described before for cartilage and tendon cells expression profile convergence in monolayer has been reported elsewhere^19^,^20^.

Dedifferentiation was confirmed by reductions in the expression of hallmarks of functionality in cartilage (*Col2a1, Agcn*) and tendon (*Tnmd, Serpinf1*). Loss of this functional synthetic profile was characterised by elevated expression of genes commonly associated with markers of mesenchymal stem cell status (*Thy1, Prnp)*^21^,^22^,^23^,^24^ and musculoskeletal developmental processes (*Pitx1, Scx, Slit3*). The homeobox gene *Pitx1*^25^, associated with specification of the hind limbs, was significantly upregulated in dedifferentiated chondrocytes; *Slit3*, homolog of *slit* (Drosophila), known to have a tendon-associated knock-out phenotype in mice^26^, was up-regulated in tenocytes in culture. In Drosophila, *slit*, secreted by tendon progenitors influences myotube migration through the ROBO receptor^27^ making this a potentially interesting marker of nascent tendon-like structures *in vitro*.

Cartilage and tendon progenitors in the limb have been defined principally by the binary expression of *Sox9* or *Scx* respectively; mesenchymal progenitor cells, although having a close temperospatial relationship diverge into cells with restricted lineage^28^. Contemporary studies suggest that cell-fate determination based upon the categorical expression of *Sox9* or *Scx* is insufficient; evidence supports the presence of Sox9^+^/Scx^+^ sub-populations of tendon and cartilage progenitors that enable the formation of transitional zones between nascent musculoskeletal tissues^2^. Consequently, temporally co-ordinated signals from numerous effector pathways define the divergent cell fates^1^. Dedifferentiated chondrocytes and tenocytes both exhibited higher expression of *Scx* relative to native tissue by qPCR. For tenocytes, *Scx* expression remained high in fibrin cultures. Expression of *Sox9*, diminished in monolayer chondrocytes and tenocytes, was significantly elevated in alginate cultures. Overall, this demonstrated the higher expression of developmental modulators of tendon (*Scx*) and cartilage (*Sox9*) in 3D culture, but an overall failure to restitute the differentiated synthetic profile. Notably, direct conversion to a chondrocytic phenotype is possible when monolayer tenocytes are exposed to *Sox9*^29^, indicating the transdifferentiation potential for cultured musculoskeletal cells.

In general, the proliferative phenotype of musculoskeletal cells in monolayer culture exhibits some of the synthetic profile of predifferentiated mesenchymal cells. Consequently, a working qualitative definition of dedifferentiated musculoskeletal cells should make reference to ‘a proliferative, pre-differentiated phenotype expressing a number of key markers of musculoskeletal progenitors’ with the caveat that with the currently available data equivalence with musculoskeletal progenitors cannot be made. Global gene expression comparisons between monolayer-expanded cells, putative adult tissue-resident somatic ‘stem cells’, and mesenchymal stem cells would rationalise this definition and clarify ambiguities associated with these cell types in differentiation studies.

Improvements to organo-typic models for cartilage and tendon have been *ad hoc* and do not represent the iterative analysis of synthetic profiles relative to native tissue. Studies often assert improvements through a limited number of differentiation markers without consideration for the gene regulatory networks that underlie the phenotypic changes. Previous studies have demonstrated the redifferentiation potential of three-dimensional constructs for both dedifferentiated chondrocytes^12^,^30^ and tenocytes^31^, but have not addressed parity with native tissue at a global gene expression level.

Although three-dimensional culture models failed to restitute the native gene expression profile instead they provide novel insights into musculoskeletal cell responses to novel environments. Common to both models was evidence of differential expression of genes associated with inflammation, differentiation, and oxidative stress. Alginate beads demonstrated both a pro-inflammatory and differentiation-associated synthetic profile with the putative osteoarthritis biomarker *Chi3l1*^32^ the most highly expressed. Similarly, in fibrin cultures markers of differentiation were not restored; *Cxcl1* and *Cox2* were found alongside expression of development genes including *Mfap5* (associated with E14.5 tendon development)^33^, *Scx,* and *Grem1* (Gremlin1)^34^.

Both three-dimensional constructs were associated with evidence of oxidative stress and hypoxia shown by the significant upregulation of *Atf4*, *Nfe2l2* (also known as *Nrf2*), and *Hif1a* by qPCR and microarray, gene ontology annotations, and Signalling Pathway Impact Analysis. Using Ingenuity^®^ Pathway Analysis mechanistic networks inferred an ‘NFE2L2-mediated oxidative stress response’. In physiological states NFE2L2 is constituitively expressed, but in conditions of oxidative stress rapid degradation by ubiquitination does not occur^35^. Consequently, NFE2L2 translocates to the nucleus, where it binds as a heterodimer with MAF proteins, or ATF4, to antioxidant responsive elements (ARE) in upstream regulatory regions of phase II antioxidant genes (*Hmox1*) to initiate their transcription.

Oxidative stress is considered a critical route to the development of dysfunctional cartilage and tendon including in osteoarthritis and tendinopathy (rotator cuff^36^, fluroquinonlone-induced tendon rupture^37^). NFE2L2 signalling and antioxidant transcripts are relevant to the protection of cartilage. Cartilage erosion is exacerbated in induced arthritis in NFE2L2 knock-out mice relative to wild-type^38^. Inhibition of histone deactylase, facilitating NFE2L2 activation, also protected against cartilage destruction in *Nfe2l2*-KO mice in two models of OA^39^. NFE2L2 is also reported as a negative regulator of chondrogenesis *in vitro*, with expression limited to proliferative and pre-hypertrophic groups in embryonic mouse tibia^40^ as such, demonstrating a differentiation role for NFE2L2 in chondrocytes. It is not clear whether the upregulation of *Nfe2l2*, in response to oxidative stress in alginate cultures, had a negative effect on chondrogenic differentiation.

In summary, the three-dimensional culture models represent elements of oxidative stress, expression of chondrogenic and tenogenic markers, expression of inflammatory chemokines and a lack of expression of functional differentiation markers. These culture models may be more representative, in qualitative terms, of a dysregulated musculoskeletal cell phenotype rather than a suitable *in vitro* proxy for cartilage or tendon.

A plethora of signalling pathways have been implicated in de- and redifferentiation transitions including TGF-beta/SMAD, PI3K/AKT, Wnt-/beta-catenin, Notch, MAPK, and ERK^14^. Similarly, the same pathways are defined as being relevant to musculoskeletal disease^41^ and limb^42^, cartilage^43^ and tendon^33^ development. Further, the genetic susceptibility to OA is known to be associated with a number of biological pathways^44^. Despite this, the core regulatory mechanisms, integration/cross-talk, and the temporal nature of these signals have not been defined. Consequently, a deeper understanding of the molecular mechanisms governing phenotypic plasticity of cartilage and tendon cells for *in vitro* models should assist with an understanding of musculoskeletal pathophysiology.

In this study known master regulators are predicted to be associated with the observed phenotypes including TGF-β (monolayer cultures) and PDGF BB (three-dimensional cultures). Using a Signalling Pathway Impact Analysis approach to understand signalling pathway perturbations this study demonstrates PI3K- and HIF1-signalling to be associated with both de- and redifferentiation; both native tendon and cartilage show activation of reference pathways for rheumatoid arthritis and systemic lupus erythematosus, whilst 3D cultures were both activated for chemokine signalling.

Although the same signalling pathways regulating *in vitro* phenotypes are associated with cartilage and tendon disease, there is insufficient evidence to assume that the same gene regulatory mechanisms are in play. Additionally, it is not clear whether de- and redifferentiation operate as a bidirectional process or which elements predominate during these transitions, although comparable intermediate regulators are predicted within mechanistic networks for both.

The study develops hypothetical mechanistic models using the predicted regulators of the observed gene expression profiles in all comparisons. These represent testable networks for future quantitative modelling to establish approaches to sustain or direct differentiation. These *in silico* models can be then iteratively tested in the established *in vitro* 3D models using the reference data provided here. Despite evidence that the upstream regulation of de- and re-differentiation is likely to be the same in both tissues there were many differences in predicted pathways between the two, most likely due to the clear difference in the complexity of the synthetic profiles arising from matrix components. Experimental approaches that reduce the complexity of the system may help establish more robust models.

Although numerous tools exist for the development of complex three-dimensional *in vitro* models in musculoskeletal biology the underlying gene regulatory mechanisms are still not understood well enough to inform the design process. Multiple obstacles to development of *in vitro* models exist, not least of which is gene and protein expression data of sufficient depth^4^. Developing robust *in vitro* models is essential for safety and efficacy studies, but also to sufficiently inform human disease. This study demonstrates that for both cartilage and tendon cells current *in vitro* standards fall short of what is required. Future experimental systems must be designed in concert with mathematical models to integrate the complex biomechanical and biochemical signalling^45^.

## Materials and Methods

### Materials

Reagents were purchased from Sigma or Life Technologies except where indicated.

### Cell culture

Tail, common calcanean (Achilles), and hind limb deep digital flexor tendons, cartilage from the coxo-femoral and femorotibial joints, and dermis from the left flank were dissected from 3 month old male F344 rats (248 ± 25.5 g) (Charles River or Harlan Laboratories), after termination in accordance with Schedule 1 of the UK Animals (Scientific Procedures) Act 1986. Animal usage for this study was approved by the University of Liverpool Animal Welfare Committee. Cells were isolated by collagenase digestion (0.2%, type 2, Worthington) for 12–18 hours at 37 °C. Tendon and dermal tissue also underwent an initial 1 hour incubation in 0.25% trypsin. Cells were seeded, subsequently split 1:2 and passaged five times in Dulbecco’s Modified Eagle’s Medium (DMEM) with GlutaMAX, supplemented with penicillin (100 U/mL), streptomycin (100 μg/mL), amphotericin B (2 μg/mL), and foetal calf serum (10%) at 37 °C and in 5% CO_2_. Chondrocytes were then seeded into alginate (1.2%) at a density of 2 × 10^6 ^cells/mL as per^46^ and grown in supplemented DMEM containing L-ascorbic acid-2-phosphate (200 μM) for two weeks. Tenocytes (7.5 × 10^5^ cells per construct) were seeded into fibrin gels as described^47^ and grown in supplemented DMEM containing L-ascorbic acid-2-phosphate (200 μM) and aprotinin (20 μg/ml) for 7–10 days until fully contracted.

### RNA isolation and microarray

Tissues and three-dimensional (3D) tendon fibrin constructs were stored directly in RNAlater (Ambion, Life Technologies) according to the manufacturer’s instructions and pulverised under N_2(l)_. Alginate beads were incubated with sodium citrate (55 mM), sodium chloride (150 mM), pH 6 for 10 minutes at 37 °C with shaking. The cell pellet was recovered by centrifugation and washed twice in PBS. Ground tissue, cell pellets and monolayer cells were incubated at room temperature in TRIzol Reagent for 10 minutes. RNA was extracted using a single-step method^48^. Following RNA resuspension in 75% ethanol, DNAse digestion and purification were performed using RNeasy spin columns (Qiagen). RNA was quantified using an ND1000 spectrophotometer (ThermoScientific) and submitted to The Genome Centre, Barts and the London Medical School, UK. RNA integrity was measured using a Bioanalyser (Agilent) and samples analysed using the RatRef-12 v1 Expression BeadChip array (Illumina).

### Gene expression and pathway analysis

Analysis of raw expression data from 36 arrays was performed using R (version 3.0.2) and the Bioconductor packages beadarray (v2.6.0)^49^ and limma. Data was pre-processed, loess-normalized and log_2_-transformed. The number of biological replicates for each condition was; native cartilage, 4; native tendon, 5; passaged chondrocytes, 8; passaged tenocytes, 8; passaged dermal fibroblasts, 3; chondrocytes in 3D alginate culture, 4; and tenocytes or chondrocytes in 3D fibrin constructs, 4. For pairwise comparisons log_2_ fold change >0.5 (fold change >1.4), ‘adjusted p-value’ (false discovery rate, FDR) <0.05 and B-statistic (log-odds ratio) >0 cut-offs were used.

Unsupervised hierarchical clustering and principal component analysis (PCA) was carried out for the most covariant genes (>0.8) using the WGCNA methodology^50^. Gene Ontology (GO) analysis was performed using the Database for Annotation, Visualization and Integrated Discovery (DAVID, https://david.ncifcrf.gov)^51^ and hypergeometric analysis using GOstats^52^. Output GO lists were rationalised using ReviGO (http://revigo.irb.hr)^53^. The ‘SimRel’ algorithm was used to calculate the semantic similarity score; allowed similarity was set to ‘Medium (0.7)’. The UniProt *Rattus norvegicus* database (2013) was used to define the search space. Only terms with a FDR <0.001 were used.

Signalling Pathway Impact Analysis (SPIA)^54^ was applied to 58 KEGG^55^ (The Kyoto Encyclopedia of Genes and Genomes, http://www.genome.jp/kegg/) *Rattus norvegicus* canonical signalling pathways in XML format. Differential expression lists consisting of Entrez gene identifiers and log_2_ fold-changes were used as the input to the SPIA pathway topology package in R, with an FDR-adjusted p-value <0.05 considered significant. SPIA uses an impact analysis technique, which makes use of both the over-representation of differentially expressed genes in a given pathway and the pathway topology, or structure, to infer perturbations to KEGG canonical signalling pathways and predict an activation state based upon the measured expression change (log_2_ fold-change). A boot-strapping process was used to define the total pathway perturbation and define a global probability value that a pathway was significantly perturbed (after FDR adjustment) in a given comparison. Both positive and negative log_2_-fold-changes from each experimental comparison was used as an input to SPIA to provide an adequate description for de- and re-differentiation transitions for each tissue.

To infer upstream master regulators of the observed gene expression profile and generate hypothesis networks the Ingenuity^®^ Pathway Analysis (IPA^®^, Qiagen Bioinformatics, Redwood City, USA, www.ingenuity.com) knowledge base and software implementing causal analysis methods^56^ were used under license. Briefly, regulators with network connections to, and the direction of regulation within, the expression dataset were scored on their likelihood of occurring more frequently than in a random model. The top upstream regulators (including: transcription factors, small molecules, endogenous chemicals, miRNAs) were defined in this study as those with: i) the smallest ‘overlap *p*-value’ – a measure of enrichment of regulated genes within a dataset using a Fisher’s Exact Test (right-tailed), and ii) the highest ‘activation *z*-score’ – the activation state of a regulator inferred from a test of the match in up- and down-regulation patterns. To build on the mechanistic networks predicted through IPA, downstream targets of transcription factors were collected from the existing gene expression dataset. Only those genes that were differentially expressed and had a direct relationship with the master regulators were chosen. To mitigate bias in the selection and addition of nodes (genes) or functional annotations to the network only those arising from earlier analysis (qPCR, SPIA, GO analysis) were included. Protein-protein association networks were defined using STRING (www.string-db.org)^57^ for *Rattus norvegicus* using medium confidence settings.

### Quantitative RT-PCR

Reverse transcription was carried out using M-MLV reverse transcriptase and random-hexamer oligonucleotides (Promega). Quantitative PCR was carried out using Takyon MasterMix Plus, ROX for SYBR Assay (Eurogentec) on a AB7300 instrument (Applied Biosystems). Amplicons were verified by melt-curve analysis and gel electrophoresis. All primers (Supplementary Data, SD20) had efficiencies over 90%. Gene expression levels were normalised to *Rps20*, shown to have the most stable expression across all sample types using the geNorm algorithm^58^, using the comparative C_t_ method^59^ on using 3–4 four biological replicates, independent of samples used for microarray gene expression profiles, with all qPCR reactions performed in triplicate. Normalised C_t_ data were converted to the linear form (2^−ΔCt^) for statistical analysis. Following Shapiro-Wilks and Levene’s tests, differences between conditions were analysed by one-way ANOVA with Games-Howell *post-hoc* test for unequal variance between groups.

## Additional Information

****Accession codes****: Raw gene expression data is available from the ArrayExpress database (www.ebi.ac.uk/arrayexpress) using the accession code: E-MTAB-4800.

**How to cite this article**: Mueller, A. J. *et al*. A systems biology approach to defining regulatory mechanisms for cartilage and tendon cell phenotypes. *Sci. Rep.* **6**, 33956; doi: 10.1038/srep33956 (2016).

## Supplementary Material

Supplementary Information

## Figures and Tables

**Table 1:** Summary of gene ontology biological process annotations derived from genes most highly expressed in each condition.

Source|Cartilage	Biological Process	Source|Tendon	Biological Process
**Native**	Immune system process	**Native**	Muscle system process
	Immune response		Muscle contraction
	Cell cycle		Immune system process
	Defense response		Skeletal muscle tissue development
			Actin filament-based process
**Monolayer (chondrocytes)**	Single-organism metabolic process	**Monolayer (tenocytes)**	Cell-redox homeostasis
	Extracellular matrix organisation		Response to endoplasmic reticulum stress
	Oxidation-reduction process		Single organism metabolic process
	Cardiovascular system development		
	Developmental process		
**Alginate cultures**	Single-organism metabolic process	**Fibrin cultures**	Translation
	Oxidation-reduction process		Metabolic process
	Lipid metabolic process		Response to oxidative stress
	Response to oxidative stress		Apoptotic signalling pathway
	Apoptotic signalling pathway		Collagen catabolic process

All functional terms are significantly enriched at FDR <0.001 and filtered for redundant terms. Native cartilage and tendon were annotated with distinct functional terms, but overlapped for immune-associated biological processes. In contrast, monolayer chondrocytes and tenocytes demonstrated frequent overlap in functional annotations (see Supplementary Data, SD2–SD7). The terms ‘response to oxidative stress’ and ‘apoptotic signalling’ were common to fibrin and alginate cultures.

**Table 2:** Differentially expressed genes selected for high expression in pairwise comparisons between native tissue and *in vitro* cultures.

Source | Cartilage	Selected genes	Source | Tendon	Selected genes
**Cartilage**	**ECM** - *Col2a1, Acan, Thbs4, Alpl, Clu, Dmp1, Ibsp, Prg2, Prg3*	**Tendon**	**Myosins | Troponins** - *Tnnc2, Myl1, Mylpf, Tnni2*
	**Cathepsins** – *Ctsk, Ctsg, Ctsc, Ctse, Ctss;*		**ECM**- *Eln, Kera, Prg4, Dpt, Vcan*
	**Wnt-signalling** – *Frzb*		**Differentiated** – Tnmd*, Serpinf1*, Mustn1*, Igfbp6, Thbs4, Myod1
	**Chemokines** – *Il18, Ccl1, Ccl5*		**Transcription factors** – *Bmp1, Bmp7, Fos*
	**Immune-associated** – *Tlr7, Defa5, Camp*		**Homeobox** – *Hopx, Tbx15*
	**CCN-family** – *Ccn2/Ctgf, Wisp1, Wisp2*		
	**Homeobox** – *Hopx, Satb1, Satb2, Hhex*		
**Monolayer (chondrocytes)**	**Mesenchymal markers** – CD90/Thy1*, Prnp*, Snai1, Twist1	**Monolayer (tenocytes)**	**Mesenchymal markers** – CD90/Thy1*, Prnp*, Twist1
	**High-expression** – *Mmp3, Tgfb2, Thbs2, Col8a1*		**High-expression** - *Pla2g7, Hmox1, Itga11, Spp1, Serpine1*
	**Wnt-signalling** – *Fzd1, Fzd2, Fzd8*		**Developmental** – Irx5, Grem1, Runx1, Slit3*
	**Homeodomain** – Pitx1*, Prrx2, Six1, Hoxa9, Hoxa10, Hoxc10, and Hoxd10		**Transcription factors** – *Cebpb, Cebpg*
	**TGF-beta signalling** – *Tgfb2, Tgfb3, Smad6, Smad7*		
**Alginate cultures**	**Oxidative stress** – Hif1a*, Atf4*, Nfe2l2*, Hmox1, Sod2	**Fibrin cultures**	**Developmental** – *Grem1, Tnn*
	**Chemokines** - *Ccl2, Ccl2, Cxcl1, Cxcl13, Cxcl16*		**Oxidative stress** – Nfe2l2*, Hif1a*, Hmox1, Sod2
	**Cytokines** - *Il6, Tnfsf15, Tnfsf13*		**Osteogenic** – *Runx1, Gpnmb*
	**Immune-mediators** – *Tlr2, Tlr3, S100a4, S100b*		**Chemokines** *– Cxcl1, Ptgs2*
	**Chondrogenesis** – *Scrg1, Id1, Bhlhb2/Dec1*		**3D-associated** *– Clu, Vegfb, Fos, Junb, Atf3*
	**Metalloproteinases** *– Mmp3, Mmp14, Adamts1*		**Metalloproteinases** *- Adamts1, Mmp11*

Threshold for differential expression was: log_2_ fold change >0.5 (fold change >1.4), adjusted *p*-value (false discovery rate, FDR) <0.05, and B-statistic (log-odds ratio) >0. Expression validation by qPCR is highlight by an asterisk (*). ECM- extracellular matrix. A complete list of differentially expressed genes passing filtering thresholds, for each pairwise comparison, are found in Supplementary Data, SD8–SD17.
